# A Comparative Evaluation of Dentogingival Tissue Using Transgingival Probing and Cone-Beam Computed Tomography

**DOI:** 10.3390/medicina58091312

**Published:** 2022-09-19

**Authors:** Gotam Das, Abdul Razzaq Ahmed, Ghazala Suleman, Abhishek Lal, Muhammad Haseeb Rana, Naseer Ahmed, Suraj Arora

**Affiliations:** 1 Department of Prosthodontics, College of Dentistry, King Khalid University, Abha 61321, Saudi Arabia; 2 Department of Prosthodontics, Altamash Institute of Dental Medicine, Karachi 75500, Pakistan; 3 Department of Restorative Dental Sciences, College of Dentistry, King Khalid University, Abha 61321, Saudi Arabia

**Keywords:** gingival biotype, esthetics, biologic width, transgingival probing, cone-beam computed tomography

## Abstract

*Background and Objective*: Gingival biotype can be assessed using a variety of invasive and non-invasive procedures, such as direct probing, transgingival probing, ultrasound-guided approaches, and, for the more sophisticated, cone-beam computed tomography. The aim of this study was to evaluate gingival biotype in relation to transgingival probing and cone-beam computed tomography (CBCT). *Materials and Methods*: This study included a total of two hundred healthy individuals. Gingival thickness was assessed and measured from the right and left maxillary central incisor teeth using CBCT and transgingival probing of the attached gingiva. The measurements were analyzed with regard to tooth type (central incisor). Linear measurements for gingival biotype were measured using both methods. Correlations and differences between measurement methods were assessed. *Results*: The mean age of study participants was 32.49 ± 8.61 years. The radiographic measurements on CBCT were 1.34 ± 0.17 mm for the right central and 1.28 ± 0.21mm for the left central. The transgingival probing measurements were 1.31 ± 0.18 for the right central and 1.22 ± 0.21mm for the left central. *Conclusion*: As per the results of this study, there is a significant positive correlation between transgingival probing and CBCT measurements of gingival biotypes.

## 1. Introduction

Gingival phenotype is a term that describes clinical differences in the morphology of gingival tissue [1]. Two types have been defined, namely thick and thin, according to the thickness of the gingivae [1,2]. Gingival phenotype is measured due to its relevance to prognosis in cases involving inflammation and trauma across several dental disciplines. A proper analysis of the soft tissue biotype is critical for the effectiveness of restorative, periodontal, and dental implant treatments, as sufficient thickness of the soft tissue can prevent negative consequences succeeding surgical operations, orthodontic therapies, and prosthodontic treatments [1,2,3,4].

Gingival biotype refers to the diameter of the faciopalatal proportions of the gingiva [1]. Gingival biotype is considered “thin” if this measurement is equal to or lesser than 1.5 mm, and it is considered “thick” if it is equal to or more than 2mm [4]. Gingival measurements, including breadth and thickness, vary significantly between individuals and are related to tooth form and shape [5]. Individuals with thin gingival tissues experience slightly higher levels of recession than those possessing wider and more robust gingiva. Most notably, the masticatory keratinized mucosa is sparse in other parts of the oral cavity, particularly the hard palate, making the covering of surgical roots by extricating connective tissue more challenging in such people [6].

Among the factors that may affect the prognosis of dental procedures, gingival biotype is a key source of concern. It has the potential to influence the end result of periodontal procedures, root planning surgeries, and the placement of dental implants. Different types of gingival biotypes react variably to inflammation as well as to surgical and restorative procedures. Consequently, identifying gingival tissue biotypes prior to treatment planning is crucial [7]. When treating patients that possess a gingival biotype that is thin, special care must be taken [8]. The value of having an appropriate biotype can be separated into two categories. Firstly, it improves initial wound covering by increasing vascularity, site protection, and tissue regeneration. Secondly, it is less susceptible to gingival/mucosal recession and mechanical discomfort, and it can form barrier that covers restorative margins [9].

Gingival thickness can be measured using a variety of invasive and non-invasive procedures, such as direct probing, transgingival probing, ultrasound-guided approaches, and, as of recently, cone-beam computed tomography (CBCT). Periodontal probing-based gingival biotype assessment is a straightforward, somewhat objective, and clinically useful approach [7,9]. Chen et al. employed a digital voltmeter to describe two types of gingival biotypes present in natural dentition: thick and thin [10]. In a study by Becker and colleagues, the authors suggested three different periodontal morphotypes: scalloped, flat, and prominent scalloping of the gingiva. When the height of the bone interproximal to the midfacial height was measured, the following results were obtained: flat = 2.1 mm, scalloped = 2.8 mm, and pronounced scalloped = 4.1 mm [11].

The utilization of ultrasonic instruments is a non-invasive modality for assessing thickness and is a reproducible procedure [12], but its disadvantages include challenges in maintaining transducer directionality [13], lack of device availability [14], and high prices. These considerations may be to blame for the device not being a standard component of the clinician’s arsenal. A simplified method for distinguishing thin gingival tissue from thick gingival tissue, based on the visibility of the periodontal probe from the gingival edge, has also been presented [15,16,17].

Recently, cone-beam computed tomography (CBCT) imaging has been used as an advanced diagnostic aid in assessments of the width of the oral hard and soft tissues [16]. Because of its greater diagnostic capacity, CBCT imaging techniques have been largely employed for scanning hard tissues. Unlike transgingival probing and ultrasonic devices, the CBCT approach provides a representation of the teeth, gingival tissues, and the remaining periodontal tissues. Furthermore, evaluations can be conducted multiple times on a single image acquired by gingival soft tissue CBCT imaging; that is not possible with other modalities. According to Fu et al. [17], CBCT gives precise measurements of the thickness of bones and labial soft tissues. He concluded that measurements obtained by CBCT might be a more objective method than direct measurements for defining the thickness of the hard and soft tissues.

In his study, Cao [11] applied three methods with respect to failing teeth to determine the width of the gingival biotype. For evaluation, he used eye inspection, probing of the periodontal tissues, and direct measurement. Before extraction, the biotype of the gingival tissues was classified, by visual inspection and evaluation with a periodontal probe, as either thick or thin. Following tooth extraction, the thickness of the gingiva was measured directly, using a tension-free caliper, to the nearest 0.1 mm [11].

CBCT can be used as a non-invasive method for assessing the biotype of gingival tissues and determining the thickness of facial gingiva and cortical bone. The purpose of this study was to assess the accuracy of CBCT in measuring gingival biotype when compared to the transgingival probing method in the aesthetic zone of maxillary teeth.

## 2. Methodology

This descriptive cross-sectional study included 200 dental implant candidates who were sent to a radiology clinic for CBCT scans. The ethical review committee of the Altamash Institute of Dental Medicine (number AIDM/ERC/06/2021/04) approved the study. Patients with at least two maxillary anterior teeth were chosen using convenience sampling, and their informed consent was obtained. Individuals possessing maxillary anterior teeth, practicing sufficient oral hygiene, without clinical symptoms of loss of attachment or inflammation, and who were systemically healthy were chosen for the study.

Patients with periodontal pockets, gingival recession, cervical abrasion, root caries, restoration, periapical disease, inflamed gingiva, tilted or rotated teeth, or fracture teeth were excluded from the study. Also excluded were women who were pregnant or nursing, patients with any history of trauma to the teeth, discoloration, or serious misalignment, and those who were taking any medication or receiving radiation treatment.

### 2.1. Clinical Examination

**Figure 1:**
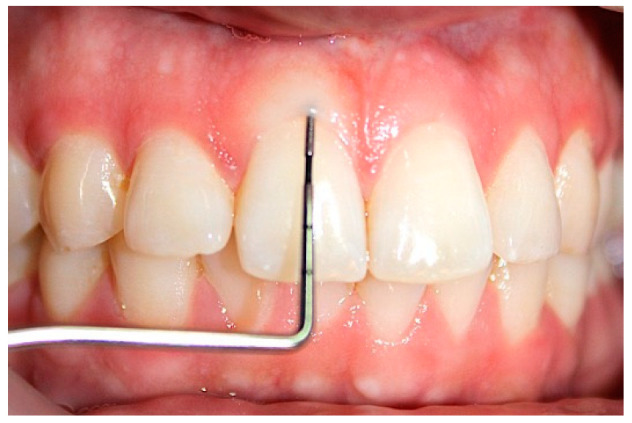
Transgingival probing by examining the gingival tissue.

Clinically, transgingival probing was used to assess gingival biotype, i.e., the thickness of the gingiva, in maxillary anterior teeth, such as both central and lateral incisors. A local anesthetic of lidocaine with adrenaline 1:200,000 was used to provide an infiltration nerve block. Following anesthesia, an endodontic K file number 20 ossessing a stopper was used for transgingival probing of connected gingival tissue at the mid-labial region, equidistant between the marginal gingiva and the mucogingival junction. The K file was inserted perpendicularly to the gingiva and penetrated until it reached the bone. The gingival thickness was determined with a vernier caliper calibrated in millimeters and accurate to one decimal point, as in Figure 1.

### 2.2. Radiographical Examination

A Hyperion X9 digital imaging system with cone-beam computed tomography (CBCT) accompanied by NNT image software (v. 4.6, desktop version) and a LCD monitor with 1280 × 1024 pixel resolution was used to examine gingival tissue biotypes and measurements of the maxillary teeth of the anterior region.

**Figure 2:**
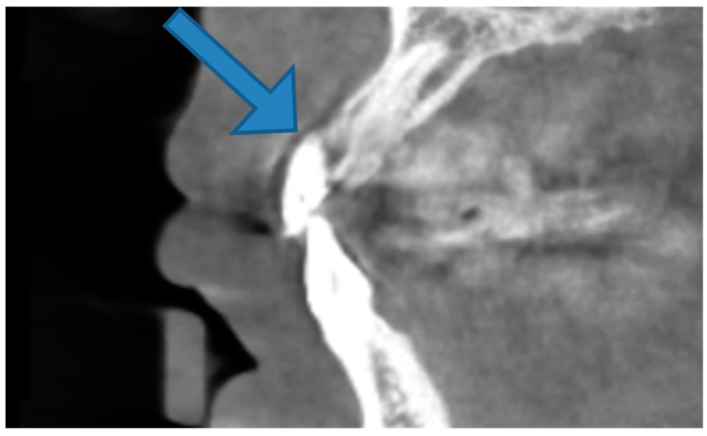
Determination of gingival thickness in CBCT (arrow).

Patients’ heads and chins were stabilized during CBCT scans. The plane of occlusion was horizontal, and the center was at the mid-sagittal plane. In the patient’s mouth, a plastic lip retractor was inserted such that the buccal mucosa and cheek did not come into contact with the teeth’s facial aspects. The patients were encouraged to maintain their tongue in the lower half of the oral cavity, as described by Khan [15]. Soft tissue cone-beam computed tomography is the name given to this technique (ST-CBCT). The soft tissue scanning mode was only used in the maxilla, with a mean exposure period of 11–12.3 s; images were taken in continuous mode at 60–65 KVp and 8–10 mA. The scan’s field of view (FOV) was 11 × 8 mm, with a resolution of 300 m. The teeth that were selected were seen in a sagittal cross-sectional view at the midline, relating to the long axis of the tooth for all measurements. When viewing a specific tooth, proper attention should be paid to ensure that the cross-sectional view, i.e., from the crown to the apex, is in one plane. CBCT images were generated and analyzed using software (Veraviewepocs 3DF40 J.Morita MFG CORP. Kyoto, Japan). Subsequently, a senior radiologist analyzed and measured the gingival thickness of the maxillary anterior teeth on a CBCT sagittal section. The thickness measurements were taken at the mesial–distal midpoint of each tooth, as shown in Figure 2. All of the data were entered into a specially developed application.

### 2.3. Statistical Analysis

The data collected were entered and analyzed using the computer application SPSS version 23. Quantitative variables, such as age and radiographic measurements obtained via CBCT, were represented in the mean and standard deviation forms. Qualitative data, such as gender and transgingival measurement method, were represented as percentages and their frequencies. A Bland–Altman plot was computed to determine the association between the clinical method (transgingival probing) and the radiographic method (CBCT) for the diagnosis of gingival biotype. *p*-value ≤ 0.05 was considered to be statistically significant.

## 3. Results

**Figure 3:**
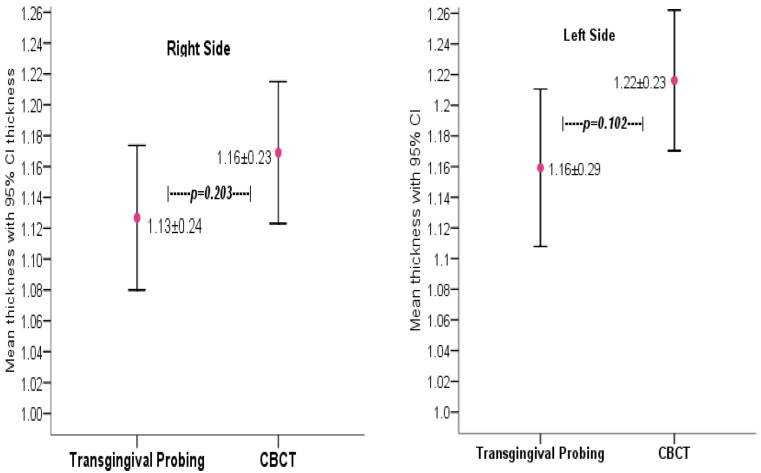
Soft tissue thickness, as measured by two methods, displayed according to the right and left sides.

A total of 200 systemically healthy subjects having maxillary central incisors and lateral incisors were included in this study to measure gingival biotype, or gingival thickness, using transgingival probing and CBCT. The difference in mean gingival thickness was not statistically significant when comparing the measurements obtained by transgingival probing and those obtained by CBCT imaging of the facial surface of teeth (*p* > 0.05), as shown in Figure 3. In addition, according to the thin and thick categories, no significant difference among methods was observed in gingival biotype thickness, as shown in Table 1.

**Figure 4:**
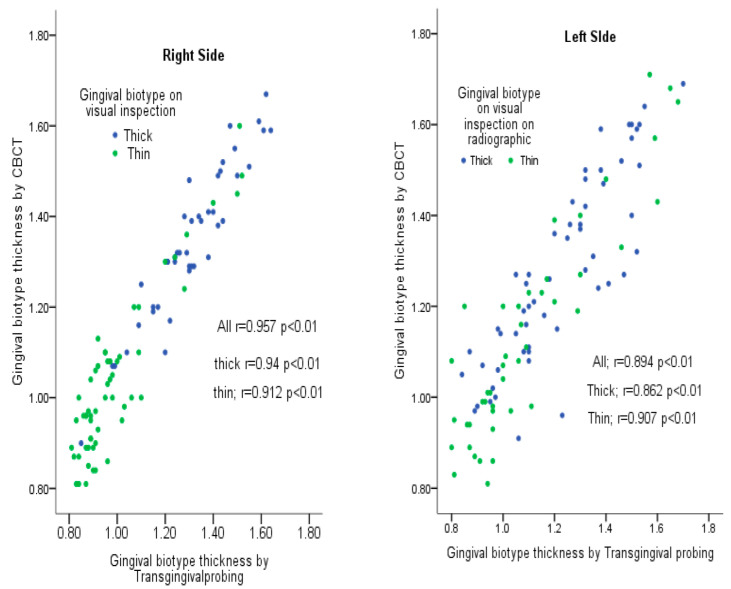
Correlation between different measurement methods.

The correlations between the measurement methods are shown in Figure 4. There was a significant positive correlation between transgingival probing and CBCT (r = 0.95; *p* < 0.01) for the right side. There was a positive and strong correlation between methods, and also for the thick and thin gingival categories (r = 0.94, *p* < 0.01 and r = 0.912, *p* < 0.01, respectively). Results were similar for the left side; a significant positive correlation between transgingival probing and CBCT was observed (r = 0.894; *p* < 0.01),as was a strong correlation for the thick and thin categories.

**Figure 5:**
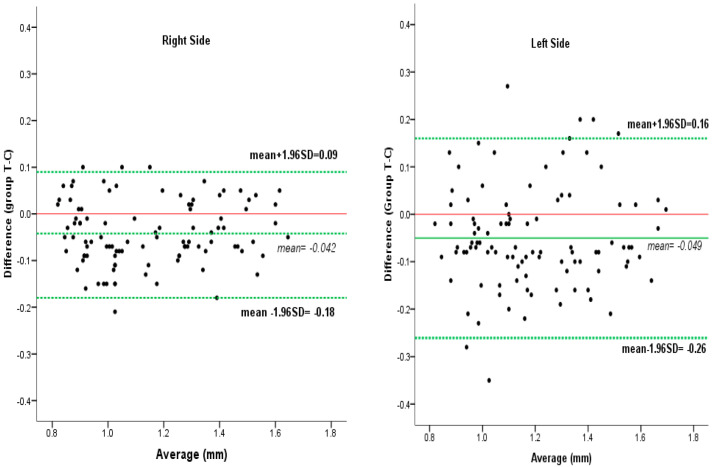
Bland–Altman plot showing the measurement agreement between methods.

Intra-class correlation was also computed to observe the relationship between measurement methods;the results showed high consistency between methods (ICC = 0.94; 95% CI: 0.91–0.96; *p* < 0.01 for the right side and ICC = 0.87; 95% CI: 0.82–0.91; *p* < 0.01 for the left side). The agreement between methods is shown in a Bland–Altman plot (Figure 5).

## 4. Discussion

The biotype of gingival tissues can be determined through visual examination (direct), the probing of periodontal tissues, and direct measurements with endodontic spreaders, endodontic files, and calipers [17]. For research and clinical purposes, the measurement of the buccolingual aspects of the gingival tissue thickness is only useful for examination purposes if the adjectives “thick” and “thin” are focused on. There are different non-invasive and invasive methods used to check gingival tissue thickness, for example, ultrasonic devices, probe transparency (Tran), and CBCT imaging [18,19,20].

The utilization of ultrasonic instruments to assess the width of gingival tissues is a non-invasive technology that has been demonstrated to be repeatable [21], but the disadvantages of this technique are the difficulty of conserving the transducer [22], definitiveness, device inaccessibility, and high costs [23]. These considerations may be to blame for the device not being part of the clinician’s conventional armamentarium. To distinguish the thin gingival biotype from the thick gingival biotype, a simplified method is used that considers the visibility of the periodontal probe from the gingival edge [24].

Recently, CBCT scans have beenused as an improved diagnostic aid for assessing the thickness of the soft and hard tissues [20]. According to Fu [17] and Aisri [25], CBCT imaging gives precise measures of the thickness of both bones and labial soft tissues. They found that CBCT dimensions may be a more objective modality than direct measurements for defining the thickness of both soft and hard tissues. To investigate the thickness of the palatal mucosa, many studies have been carried out using transgingival probing, but only a few studies have been conducted that utilized soft tissue CBCT to assess the thickness of facial gingival tissue [4,8,26]. In this study, we evaluated the association between the thickness of the soft tissues of the teeth in the maxillary anterior region and the thickness of the gingival biotype, with the help of soft tissue CBCT and transgingival probing.

For this study, the age of participants ranged from 18 to 50 years, with a mean age of 35.13 ± 7.75 years. Most of the participants (28 individuals, or 70%) were between the ages of 18 and 40. Out ofa total of 40 participants, 22 (55%) were females, and 18 (45%) were males, for a ratio of 1.2:1 between females and males. The correlation between transgingival probing and CBCT assessment of gingival biotype had a Spearman’s correlation coefficient of 0.985 and a *p*-value of 0.0001; thus, it is statistically significant, with an r value of 0.401.10. Similarly, El Khalifa et al. discovered a substantial positive association between transgingival probing and CBCT assessments of gingival biotypes.

To date, there has been no specific definition of how a thick gingival biotype differs from a thin gingival biotype. Amongst the possible causes, it is noted that gingiva thickness is measured at various vertical levels. Previously, intrusive procedures were utilized to evaluate gingival thickness; direct measurement [26] was used but had several disadvantages, including an invasive approach, the lack of repeatability and precision, inappropriate angles, and the level of pressure. To circumvent such constraints, non-invasive approaches, such as ultrasonic devices [27] and cone-beam computed tomography [28], were developed; however, such modalities are technique-sensitive and have high costs. The accuracy of several techniques, including manual evaluation by the utilization of calipers after tooth extraction [29], measurement using a syringe with an endodontic depth marker, and CBCT radiography, is limited by the absence of reference objects. Recently, a technique was developed that was a modified radiographic technique [30] presented by Alpiste-Illueca [31], who discovered that crown width to crown length ratio and gingival width are contrasting morphometric measurements that can serve as replacement dimensions to predict the thickness of the gingival biotype located on the cementoenamel junction.

Gürlek et al. [29] provided a simple technique for determining periodontal type that relies on the visibility of free gingival tissue while gingival grooves in the teeth are probed. The most frequently used approach for distinguishing thin and thick gingival biotypes is the visual evaluation of the visibility of the periodontal probe through the sulcus. If the periodontal probe can be seen through the gingival edge or sulcus, the gingival tissue biotype is characterized as thin. The capacity of gingival tissue to hide the color of any underlying material is required for producing attractive outcomes, particularly with regard to implants and restorative dentistry, and subgingival metals are extensively employed for this reason [31]. The simplest technique for detecting a thin gingival biotype is to use a periodontal probe (metal in nature) in the sulcus to measure gingiva width; the tip of the periodontal probe appears to be transparent through the gingiva [32]. Periodontal probing methods are regularly used during periodontal and implant treatments because they are less invasive than alternatives [13,20,33].

Both hard and soft tissues can be seen and measured with CBCT. Several authors observed that CBCT measures both soft and hard tissue with reliability and accuracy. They noted that CBCT images might be a more objective means of determining hard and soft tissue thickness than direct measurements [28,31,33]. CBCT offers a more accurate image of the tooth, gingiva, and other periodontal structures compared to ultrasonic devices and transgingival probing. Furthermore, the dimensions of a particular tooth may be measured several times with the same image acquired by ST-CBCT, which is not possible with other techniques [33].

Stein et al. [34] conducted a comparison analysis with a total of 60 participants and discovered links between the buccal bone and gingival tissue width. Nonetheless, the contrast in their study was not conducted at the same level. Instead, the gingival biotype thickness was assessed supracrestally, whereas the thickness of the bone was measured posterior to the alveolar crest. In comparison, La Rocca et al. [35] found no noteworthy link between the outcomes of CBCT imaging and transgingival probing in an in vivo investigation of 90 maxillary teeth, despite the fact that the comparison in their study was not conducted at an equivalent level. Despite such contradictory findings, and in spite of our study’s small sample size, we found a strong positive connection between transgingival probing and CBCT measurements of gingival biotypes.

## 5. Conclusions

As per the findings of our study, there was a considerable positive relationship between transgingival probing and CBCT measurements of gingival biotypes. As a result, we urge that CBCT imaging be utilized to measure hard and soft tissue thickness; in addition, we recommend that gingival biotype be defined in all periodontal disease patients in order to deliver the predicted restorative and surgical treatment outcomes.

## Figures and Tables

**Table 1:** Comparison between methods of mean gingival biotype thickness, according to the thick and thin categories and measured on the right and left sides.

Thickness	Thick		*p*-Value	Thin		*p*-Value
	Transgingival Probing	CBCT		Transgingival Probing	CBCT	
**n**	43	43		57	57	
Right	1.31 ± 0.18	1.34.99 ± 0.17	0.429	0.99 ± 0.17	1.04 ± 0.18	0.130
**n**	56	56		44	44	
Left	1.22 ± 0.21	1.28 ± 0.21	0.133	1.09 ± 0.25	1.14 ± 0.28	0.37

## Data Availability

Not applicable.
